# Chemical Composition and Synergistic Antimicrobial Activity of *Hypericum perforatum* and *Achillea millefolium* Essential Oils Against Wound-Associated Microorganisms

**DOI:** 10.3390/molecules31101594

**Published:** 2026-05-10

**Authors:** Daniela Bordea, Alina L. Nistor, Liana Claudia Salanţă, Teodora E. Coldea, Ancuța M. Rotar, Oana M. Grigor, Rodica Vârban, Emese Gal, Carmen R. Pop

**Affiliations:** 1 Department of Environmental Engineering and Protection, Faculty of Agriculture, University of Agricultural Sciences and Veterinary Medicine Cluj-Napoca, 400372 Cluj-Napoca, Romania; daniela.bordea@usamvcluj.ro; 2 Department of Food Science, Faculty of Food Science and Technology, University of Agricultural Science and Veterinary Medicine, 400372 Cluj-Napoca, Romania; alina.nistor@usamvcluj.ro (A.L.N.); liana.salanta@usamvcluj.ro (L.C.S.); anca.rotar@usamvcluj.ro (A.M.R.); oana-maria.grigor@usamvcluj.ro (O.M.G.); 3 Department of Food Engineering, Faculty of Food Science and Technology, University of Agricultural Sciences and Veterinary Medicine Cluj-Napoca, 400372 Cluj-Napoca, Romania; teodora.coldea@usamvcluj.ro; 4 Department of Crop Science, Faculty of Agriculture, University of Agricultural Science and Veterinary Medicine, 400372 Cluj-Napoca, Romania; rodica.varban@usamvcluj.ro; 5 Faculty of Chemistry and Chemical Engineering, Babeș-Bolyai University, 11 Arany János Street, 400028 Cluj-Napoca, Romania; emese.gal@ubbcluj.ro

**Keywords:** antimicrobial synergy, fractional inhibitory concentration index (FICI), essential oils combination, wound infection, chemical profile

## Abstract

Wound-associated infections persist as a major global health concern, particularly in the context of increasing antimicrobial resistance and reduced efficacy of conventional therapies. Essential oils (EOs) obtained from medicinal plants represent promising alternatives due to their antimicrobial and wound-healing properties. This study evaluated the chemical composition, antimicrobial activity, and interaction effects of *Hypericum perforatum* (HP) and *Achillea millefolium* (AM) EOs, tested individually and in fixed-ratio combinations. Chemical profiling by GC–MS revealed that HP EO is dominated by caryophyllene (20.74%) and β-thujone (18.47%), while AM EO is characterized by aromadendrene (19.12%), caryophyllene (12.97%), and chamazulene (10.13%). Antimicrobial activity was assessed against wound-associated microorganisms using MIC and MBC/MFC assays, and interactions were assessed by the fractional inhibitory concentration index (FICI) and heatmap analysis. The results displayed higher susceptibility of Gram-positive bacteria, particularly *Staphylococcus epidermidis*, with MIC values as low as 0.56 µL/mL in EO combinations. Synergistic effects were observed exclusively for *S. epidermidis* in mixtures enriched in HP EO (60:40 and 70:30; FICI = 0.34), while Gram-negative bacteria and *Candida albicans* exhibited predominantly indifferent responses. These findings indicate that optimized EO combinations may enhance antimicrobial efficacy and support their potential application in wound management.

## 1. Introduction

Wound-associated infections persist as a major global health concern, exacerbated by the rapid emergence of antimicrobial resistance (AMR) and the declining efficacy of conventional antibiotics [1]. Chronic and acute wounds are predominantly colonized by opportunistic and multidrug-resistant pathogens, such as *Staphylococcus aureus* and *Pseudomonas aeruginosa*, which significantly compromised the wound healing process and elevate the risk of serious clinical complications [2]. The growing limitations of current antimicrobial therapies have intensified the search for alternative strategies capable of both controlling microbial infections and promoting tissue regeneration [3].

In this context, medicinal and aromatic plants have gained increasing attention as valuable sources of bioactive compounds [4]. Essential oils, complex arrays of bioactive volatile compounds such as monoterpenes and sesquiterpenes, exhibit broad-spectrum antimicrobial, antioxidant, and anti-inflammatory activities, which are particularly relevant in wound healing applications [5]. Their multi-target mechanisms of action make them viable therapeutic alternatives against multidrug-resistant microorganisms while reducing the probability of resistance development [1].

The genus *Hypericum* L. (*Hypericaceae*) comprises over 500 taxa worldwide, classified into 36 taxonomic sections [6]. Among these, *Hypericum perforatum* L. (HP), commonly known as St. John’s Wort is the most widely cultivated species [7]. This plant has been used since ancient times as a traditional medicinal remedy for wounds, burns, inflammatory diseases, gastrointestinal and biliary disorders, as well as various dermatological conditions [8,9]. The essential oil (EO) obtained from HP has attracted considerable scientific interest due to its antimicrobial, antioxidant, antifungal, and antidermatophytic activities [10]. It is frequently used in the treatment of superficial wounds, abrasions, sunburn, dermatitis, and eczema, mainly due to its anti-inflammatory properties and its capacity to promote tissue regeneration and repair [11].

From a chemical standpoint, HP EO represents a complex mixture of volatile compounds, predominantly monoterpenes and sesquiterpenes. Phytochemical analyses have identified numerous constituents, among which aliphatic hydrocarbons constitute the major fraction, followed by sesquiterpene hydrocarbons and oxygenated terpenes [12]. These compounds are regarded as the primary contributors to the observed antimicrobial and wound-healing activities.

Another plant of significant interest is *Achillea millefolium* L. (AM), named yarrow, a member of the *Asteraceae* family [13]. In traditional medicine, AM has been extensively employed for wound healing and for the treatment of inflammatory, gastrointestinal, and dermatological disorders [5,14,15]. The EO extracted from its flowers and leaves is characterized by a high content of monoterpenes, including eucalyptol, camphor, α-terpineol, β-pinene, and borneol [16]. Due to this bioactive profile, AM EO exhibits notable antibacterial and antifungal activities, suggesting potential applications in the prevention and treatment of wound infections [3]. Furthermore, experimental studies have demonstrated that AM EO can enhance wound healing by promoting collagen synthesis, reducing inflammatory responses, and accelerating tissue regeneration [2].

Recent research has highlighted that combining EOs may represent an effective strategy to overcome microbial resistance. Such combinations can lead to synergistic effects, resulting in enhanced antimicrobial activity, reduced effective concentrations, and improved efficacy against complex microbial communities, including biofilm-forming pathogens [1,17]. Notably, the synergistic interactions between EOs and antimicrobial agents have been proved to significantly reduce minimum inhibitory concentrations and enhance bacterial eradication, particularly in resistant strains [17]. This approach is particularly relevant in wound environments, where polymicrobial infections and resistant pathogens frequently limit the effectiveness of single-agent therapies and contribute to delayed healing processes [2].

Although both HP and AM have been individually investigated for their antimicrobial and wound-healing properties, data regarding their combined antimicrobial effects and potential synergistic interactions remain limited. Remarkably, clinical findings indicate that formulations containing extracts of both species can significantly reduce inflammation and improve healing outcomes, supporting their complementary therapeutic potential [18].

Accordingly, the aim of the present study is to evaluate the in vitro antimicrobial activity and synergistic effects of EOs obtained from *Achillea millefolium* (AM) and *Hypericum perforatum* (HP), both individually and in combination, against wound-associated microorganisms. Additionally, the chemical composition of the EOs was characterized using gas chromatography–mass spectrometry (GC–MS) to establish potential correlations between phytochemical profiles and antimicrobial efficacy.

## 2. Results

### 2.1. GC-MS Chemical Profiling

The hydrodistillation of dried aerial parts yielded essential oils with extraction efficiencies of 0.12% (HP) and 0.40% (AM), calculated on a dry weight basis.

The volatile composition of HP EO was determined by GC-MS analysis, which revealed a complex mixture of terpene derivatives, oxygenated compounds and aliphatic hydrocarbons. The relative abundance of the detected constituents was expressed as the percentage of the total chromatographic peak area. The identification of the volatile compounds was achieved by comparing the obtained mass spectra with those from the NIST spectral libraries and by considering their retention times.

**Figure 1:**
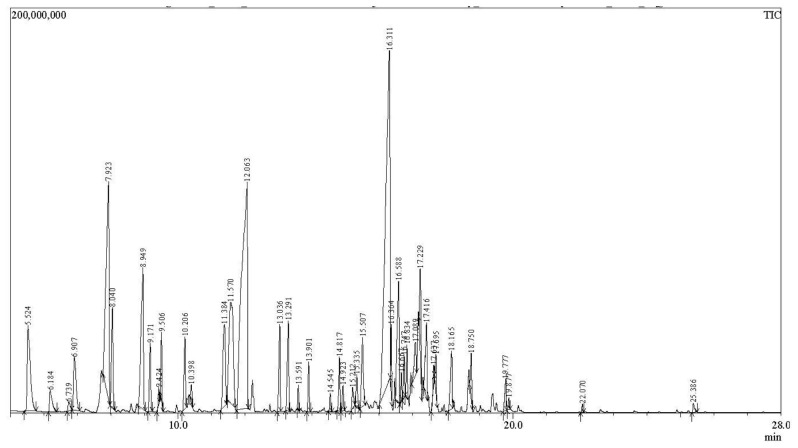
Chromatograms (TIC, total ion chromatogram) of GC-MS analysis of volatiles from *Hypericum perforatum* EO.

**Figure 2:**
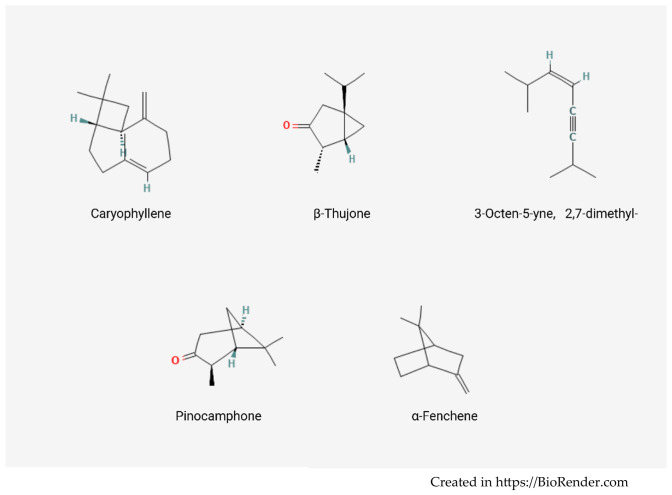
Chemical structures of the main volatile compounds of *Hypericum perforatum* EO.

The chromatographic profile (Figure 1) indicated that the volatile fraction of HP EO is largely dominated by terpene-derived compounds. The volatile profile was characterized by the predominance of a limited number of compounds with relatively high abundance. The most abundant constituents identified in the analyzed samples were caryophyllene (20.74%) and β-thujone (18.47%), followed by 3-octen-5-yne, 2,7-dimethyl- (9.03%), pinocamphone (6.36%), and α-fenchene (4.87%). Collectively, caryophyllene and β-thujone represented approximately 39% of the total volatile fraction, underscoring their predominant contribution to the overall chemical profile of the plant. In Figure 2 is presented the chemical structure of the main compounds identified.

Caryophyllene is a sesquiterpene widely reported in essential oils of aromatic and medicinal plants and is known for its woody and spicy aroma notes, as well as for its potential biological activities, including anti-inflammatory and antimicrobial effects. β-Thujone, an oxygenated monoterpene, is similarly regarded as a significant aroma-active compound imparting herbaceous and camphoraceous notes to plant-derived extracts.

Among the compounds present at moderate concentrations included 2,3,3-trimethyl-octane (3.80%), β-farnesene (3.50%), and a cyclohexene derivative (3.05%) were noteworthy. Monoterpene hydrocarbons such as β-myrcene (2.24%) and α-pinene (1.96%) were also detected among the volatile constituents.

The identified volatile compounds and their relative abundances are presented in Table 1, with the reported values representing the mean of two independent GC-MS determinations. Overall, the obtained results indicate that the volatile profile of HP EO is largely characterized by terpene derivatives, which are widely recognized as key contributors to both the aroma and biological properties of aromatic and medicinal plants.

To facilitate a systematic overview of the chemical composition of the volatile fraction, the identified compounds were classified into several chemical groups based on their structural characteristics, including sesquiterpenes, monoterpenes, oxygenated monoterpenes, aliphatic hydrocarbons and other oxygenated compounds.

Among these groups, sesquiterpenes represented the dominant chemical class (35.45%), highlighting the important role of these compounds in shaping the volatile fingerprint of *Hypericum perforatum*. Representative constituents of this class included caryophyllene (20.74%), which was the most abundant compound detected in the analyzed samples, together with β-farnesene (3.50%), α-bourbonene (1.91%), and α-amorphene (1.55%). Sesquiterpenes are frequently reported as major constituents of plant essential oils and are typically associated with woody, balsamic and slightly spicy aromatic notes.

Oxygenated monoterpenes represented a substantial proportion of the volatile fraction (30.92%), constituting an important class of aroma-active compounds in numerous aromatic plants. In the present study, this group was mainly represented by β-thujone (18.48%) and pinocamphone (6.35%), which together accounted for most of the oxygenated monoterpene fraction. These compounds are known to exert a pronounced influence on the sensory profile of plant extracts and may additionally contribute to their biological activity.

Monoterpene hydrocarbons accounted for 10.92% of the total volatile fraction. The most representative compounds within this group were α-fenchene (4.87%), β-myrcene (2.24%), α-pinene (1.96%), and cis-β-ocimene (1.35%), compounds typically associated with fresh, resinous and slightly citrus-like aroma characteristics.

In addition, aliphatic hydrocarbons (alkanes) represent 9.81% of the total identified compounds. Among them, 2,3,3-trimethyl-octane (3.80%), 2-methyldodecane (1.96%), and undecane (1.49%) were the most notable constituents. Although these compounds generally contribute less to aroma compared with terpene compounds, they are frequently detected as minor components of plant volatile fractions. Finally, other oxygenated compounds, including esters, alcohols and diterpenoid derivatives, accounted for 19.38% of the total volatile composition, further contributing to the chemical complexity of the volatile profile.

Overall, the predominance of sesquiterpenes and oxygenated monoterpenes indicates that the volatile fraction of HP is largely governed by terpene biosynthesis pathways, which are commonly associated with the characteristic aroma and biological properties of aromatic and medicinal plants. The results highlight that the volatile composition of HP EO is dominated by terpene compounds. The abundance and structural diversity of the terpene constituents identified are likely to play a central role in determining both the aromatic profile and the functional properties of this medicinally relevant species.

**Figure 3:**
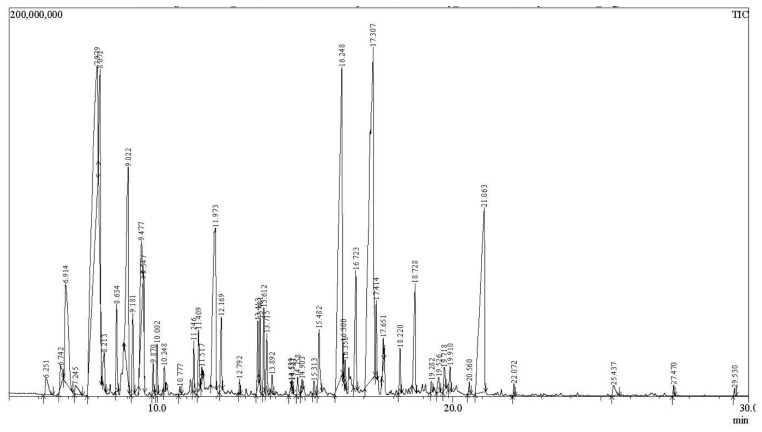
Chromatograms (TIC, total ion chromatogram) of GC-MS analysis of volatiles from *Achillea millefolium* EO.

The volatile composition of AM EO obtained by hydrodistillation was investigated by GC-MS analysis (Figure 3), revealing a complex mixture of terpene-derived metabolites and other volatile constituents. The relative abundance of each detected compound was expressed as a percentage of the total chromatographic peak area, and compound identification was performed by matching the obtained mass spectra against the NIST spectral library in conjunction with retention time data. All analyses were conducted in duplicate with reported values represent the mean of two independent GC-MS determinations.

**Figure 4:**
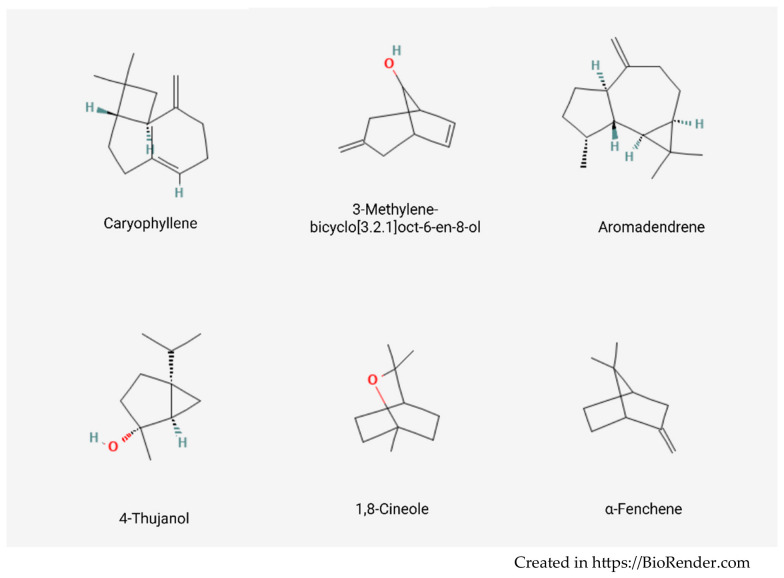
Chemical structure of the main volatile compounds of *Achillea millefolium* EO.

A total of 52 volatile constituents were detected, reflecting the chemical complexity characteristic of aromatic and medicinal plant essential oils. The volatile fraction was distinguished by the presence of several major constituents occurring at notably high proportions, as detailed in Table 2. The most abundant compound identified was aromadendrene (19.12%), followed by caryophyllene (12.97%), 3-methylene-bicyclo [3.2.1] oct-6-en-8-ol (11.41%), and chamazulene (10.13%). Other compounds detected in notable amounts included 4-thujanol/sabinene hydrate (6.27%), 1,8-cineole (5.83%), and α-fenchene (4.28%). The chemical structures of these compounds are presented in Figure 4. Additional constituents present in moderate proportions were 3-octen-5-yne, 2,7-dimethyl-(E) (3.19%), 2-carene (2.38%), cis-α-bisabolene (2.21%), and caryophyllene oxide (2.20%).

The predominance of terpene-derived metabolites observed in the present study is consistent with the chemical composition typically reported for AM EO, in which sesquiterpenes and oxygenated monoterpenes are frequently described as the dominant chemical classes within the volatile fraction of this species.

One of the characteristic constituents detected in the analyzed samples was chamazulene (10.13%), a well-established marker compound of AM EO. Chamazulene is not naturally present in intact plant tissues but is formed during the thermal processing of plant material through the degradation of the sesquiterpene lactone matricin [19]. This transformation may occur during extraction techniques involving heating, including hydrodistillation extraction. Chamazulene is widely recognized for its anti-inflammatory and antioxidant properties and is considered an important bioactive constituent of AM EO.

Other major compounds identified in the present study, such as caryophyllene and 1,8-cineole, have likewise been extensively reported in the volatile fractions of *Achillea* species. Caryophyllene, a sesquiterpene hydrocarbon, is well documented for its antimicrobial and anti-inflammatory activities, while 1,8-cineole contributes to both the characteristic aroma and the biological properties of numerous medicinal plants. The occurrence of these compounds may therefore underpin the biological potential traditionally associated with *Achillea millefolium* extracts.

The chemical profile observed in this study suggests that the analyzed *A. millefolium* sample may correspond to a chamazulene/caryophyllene-type volatile profile. Nevertheless, it is well documented that the volatile composition of AM EO is subject to considerable variation depending on geographical origin, climatic conditions, plant developmental stage and extraction technique, factors that frequently give rise to distinct chemotypes within the species.

To facilitate a systematic overview of the chemical composition of the volatile fraction, the identified compounds were classified into several chemical groups based on their structural characteristics, including monoterpene hydrocarbons, oxygenated monoterpenes, sesquiterpene hydrocarbons, oxygenated sesquiterpenes, esters and other aliphatic compounds.

Sesquiterpene hydrocarbons constituted the dominant chemical class, reflecting the substantial contribution of this group to the volatile fingerprint of AM EO. Representative constituents of this class included aromadendrene (19.12%), which was the most abundant compound detected in the analyzed samples, followed by caryophyllene (12.97%) and chamazulene (10.13%). Additional sesquiterpene hydrocarbons were identified in lower amounts, including cis-α-bisabolene (2.21%), β-bourbonene (1.28%), γ-elemene (0.96%), β-sesquiphellandrene (0.33%), germacrene D (0.23%), α-cubebene (0.21%), D-amorphene (0.29%), and δ-elemene (0.06%).

Oxygenated monoterpenes represented a substantial proportion of the volatile fraction, constituting an important class of aroma-active compounds in many aromatic plants. In the present study, this group was mainly represented by 3-methylene-bicyclo [3.2.1] oct-6-en-8-ol (11.41%), 4-thujanol/sabinene hydrate (6.27%), and 1,8-cineole (5.83%). Other compounds belonging to this class included α-terpineol (1.10%), chrysanthemol (0.99%), yomogi alcohol (0.76%), camphor (0.66%), linalool (0.31%), pinocarvone (0.17%), and p-menth-2-en-1-ol (0.14%).

Monoterpene hydrocarbons accounted for a smaller portion of the volatile profile. The most representative compounds in this group included α-fenchene (4.28%), 2-carene (2.38%), α-terpinolene (1.35%), and cis-β-ocimene (1.26%), while santolina triene (0.63%), α-thujene (0.55%), and camphene (0.38%) were detected in minor amounts.

In addition, esters were represented by several compounds, including geranyl acetate (1.11%), 4-thujen-2-yl acetate (0.90%), bornyl acetate (0.80%), and (−)-trans-myrtanyl acetate (0.65%), together with smaller quantities of α-terpinyl acetate (0.28%), pinocarvyl acetate (0.19%), and neryl acetate (0.16%).

Collectively, oxygenated sesquiterpenes such as caryophyllene oxide (2.20%), β-nerolidol (0.63%), α-cadinol (0.43%), δ-cadinol (0.36%), spathulenol (0.37%), and epiglobulol (0.15%) were also detected. Overall, the predominance of sesquiterpene hydrocarbons and oxygenated monoterpenes indicates that the volatile fraction of AM EO is largely governed by terpene biosynthesis pathways, which are characteristic of aromatic and medicinal plants.

### 2.2. Antimicrobial Activity of EOs

The antimicrobial activity of HP and AM EOs, evaluated individually and in combination, demonstrated a clear dependence on both microbial type and oil ratio. Minimum inhibitory concentration of both individual and in combination EOs are presented in Table 3, while minimum bactericidal/fungicidal concentration are reported in Table 4.

Overall, Gram-positive bacteria displayed greater susceptibility than Gram-negative strains. Among the microorganisms tested, *S. epidermidis* exhibited the highest sensitivity, with remarkably low MIC values reaching 0.56 μL/mL and corresponding MBC values of 0.56–1.17 μL/mL for the 60:40 and 70:30 mixtures, indicative of a pronounced bactericidal effect. *S. aureus* also showed enhanced susceptibility in combined formulations, with MIC values decreasing from 22.68 μL/mL for HP EO alone to 5.14 μL/mL in all mixtures. In contrast, *S. pyogenes* displayed moderate sensitivity, with relatively stable MIC values (10.80 μL/mL), although certain combinations yielded reductions in bactericidal concentrations. Gram-negative bacteria were generally more resistant, which is consistent with the presence of an outer membrane that restricts the penetration of hydrophobic compounds. *P. aeruginosa* was the most resistant strain, with constant MIC values of 22.68 μL/mL and high MBC values (47.62 μL/mL) across all samples. *E. coli* and *K. pneumoniae* showed moderate susceptibility, with improved activity observed in several mixtures, where MIC values decreased to 10.80 μL/mL. These findings suggest a partial enhancement of antibacterial activity through oil combination, likely due to additive or synergistic interactions between bioactive constituents. The antifungal activity against *C. albicans* was moderate, with MIC values ranging from 10.80 to 22.68 μL/mL. The EO of AM exhibited the strongest antifungal effect (MIC = 10.80 μL/mL), while combinations did not significantly enhance activity and, in some cases, resulted in higher fungicidal concentrations, indicating predominantly fungistatic effects.

## 3. Discussion

### 3.1. GC-MS Chemical Profiling

The identification of volatile compounds was performed using GC–MS, a widely recognized and reliable technique for essential oil analysis. Compound identification was based on mass spectral matching with NIST and WILEY libraries and further supported by retention indices calculated relative to n-alkanes. This combined approach ensures a high level of accuracy and reproducibility in the characterization of complex volatile mixtures.

The volatile profile obtained in this study is broadly consistent with previously reported compositions of HP EO and volatile extracts. Literature data indicate that EOs of these species are typically dominated by monoterpene and sesquiterpene hydrocarbons, particularly α-pinene, β-pinene, (E)-caryophyllene and germacrene D, which are commonly described as major constituents of the genus [6,20]. Similar compositional patterns have been reported for HP EO, with β-caryophyllene and other terpenes frequently identified as major constituents, highlighting the important contribution of sesquiterpene hydrocarbons to its volatile fingerprint [11,21]. Studies on *Hypericum* species have likewise emphasized considerable chemical diversity, with sesquiterpenes and oxygenated terpenoids often representing dominant classes [7,22]. Nevertheless, it is well documented that the chemical composition of volatile fractions in *Hypericum* species may vary considerably depending on several factors, including geographical origin, environmental conditions, plant phenological stage and extraction technique [6], factors that may account for the compositional differences observed across studies.

Several volatile constituents identified in HP EO have been associated with important biological activities. Terpenes such as α-pinene, β-pinene and β-caryophyllene are known to exhibit antimicrobial, antioxidant and anti-inflammatory properties, which may contribute to the biological potential of *Hypericum* extracts [11]. Caryophyllene, a major sesquiterpene widely distributed in essential oils from spice, food and medicinal plants, has been associated with antimicrobial, antibiofilm, anti-inflammatory, antioxidant and cytoprotective activities. Its biological effects have been linked to activity against Gram-positive bacteria, modulation of pro-inflammatory mediators and oxidative stress, supporting its multifunctional biological potential [23]. In addition to volatile terpenes, HP is known to contain several classes of bioactive compounds, including phloroglucinols, naphthodianthrones, flavonoids and phenolic acids, which are responsible for the diverse pharmacological properties of this medicinal plant [9].

The volatile profile obtained in the present study is generally consistent with previously reported compositions of AM EOs and volatile extracts. Several phytochemical investigations have shown that EOs of *Achillea* species are typically dominated by terpene compounds, particularly sesquiterpenes and oxygenated monoterpenes [3]. Comparable compositional patterns were reported by Farhadi et al. [5], who identified terpene-derived metabolites such as caryophyllene, chamazulene and oxygenated monoterpenes among the dominant constituents of AM EO. Similarly, the study conducted by Jangjoo et al. [14] highlighted the occurrence of several terpene compounds as major constituents of *Achillea* EOs, further confirming the important contribution of these metabolites to the volatile fingerprint of the species. However, it is well documented that the chemical composition of volatile fractions in *Achillea* species may vary considerably depending on several factors, including geographical origin, environmental conditions, plant phenological stage and extraction method [24]. Several volatile constituents identified in AM EO have likewise been associated with significant biological activities. Terpene compounds such as caryophyllene, 1,8-cineole and chamazulene are known to exhibit antimicrobial, antioxidant and anti-inflammatory properties, which may contribute to the biological potential of *Achillea* extracts [5]. β-Thujone has been associated with antimicrobial, antiviral and immunomodulatory activities and has been reported to contribute to the antibacterial activity of thujone-containing essential oils. In addition, its modulation of GABA-gated chloride channels suggests broader biological activity beyond antimicrobial effects [25]. Chamazulene, which represented one of the major constituents identified in the present study, is widely recognized for its anti-inflammatory activity and is responsible for the characteristic blue coloration of some *Achillea* EOs. It has been reported in *Matricaria recutita* and *Artemisia arborescens* and has been associated with antioxidant and anti-inflammatory activities through free radical scavenging, inhibition of lipid peroxidation and modulation of inflammatory mediators such as TNF-α and IL-6. Hepatoprotective and pro-apoptotic effects have also been described, supporting its broader biological potential and possible contribution to the activity of chamazulene-rich essential oils [26]. Beyond volatile terpenoids, AM EO is known to contain several classes of bioactive compounds, including flavonoids, phenolic acids, and sesquiterpene lactones, which contribute to the diverse pharmacological properties of this medicinal plant [3,14].

**Figure 5:**
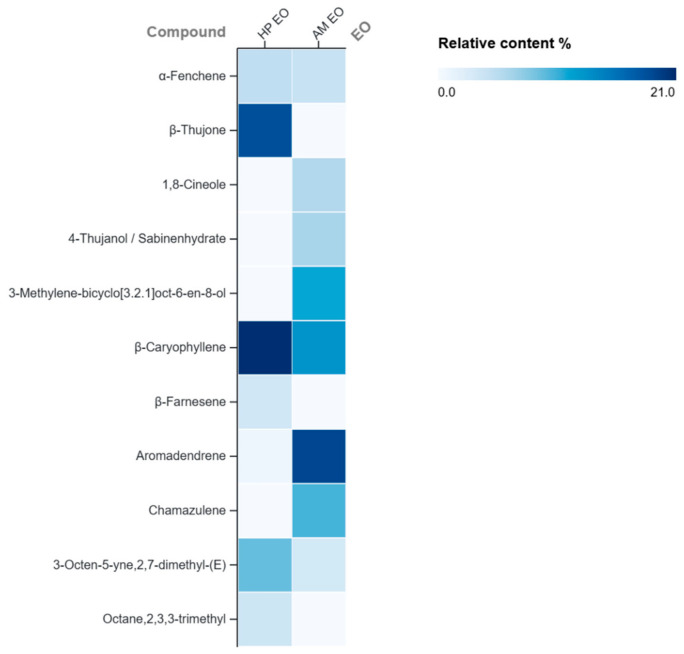
Comparative heatmap of major compounds identified in HP and AM EOs.

The comparative heatmap (Figure 5) highlighted both shared and distinct chemical features of the two essential oils. Both EOs showed high abundance of β-caryophyllene, HP was characterized by higher β-thujone content and AM EO by greater abundance of aromadendrene, chamazulene and oxygenated monoterpenes. These compositional differences indicate complementary chemical profiles that may contribute to the strain-dependent responses and synergistic interactions observed in the EOs combinations. Overall, the data support that the biological activity is likely driven by cooperative interactions within the whole phyto-complex rather than by individual constituents alone.

### 3.2. Antimicrobial Activity

Antimicrobial activity was assessed using the resazurin-based microdilution method, which is a sensitive and widely applied technique for determining minimum inhibitory concentrations of essential oils. Subsequent subculturing for the determination of MBC and MFC values enabled differentiation between bacteriostatic/fungistatic and bactericidal/fungicidal modes of action, providing a reliable and comprehensive in vitro assessment of antimicrobial efficacy. In Supplementary Material are presented images of MIC and MBC results for some strains.

Comparison between individual oils and their combinations revealed a ratio-dependent improvement in antibacterial activity, particularly against Gram-positive bacteria. The mixtures containing higher proportions of HP (60%:40% and 70%:30%) showed the most pronounced effects, suggesting synergistic interactions that enhance microbial inhibition. In many cases, MBC values were approximately two-fold higher than MIC values, indicating a bactericidal mode of action; however, larger differences observed for certain strains point to bacteriostatic effects.

Although the EOs exhibited lower antimicrobial potency compared to the reference agents (gentamicin and ketoconazole), their biologically relevant activity supports their potential use in wound management applications. The pronounced efficacy against Gram-positive bacteria, which are frequently implicated in skin and soft tissue infections, highlights their suitability for topical formulations. Moreover, the enhanced activity observed for specific combinations suggests that optimized ratios may improve antimicrobial performance while potentially reducing required concentrations. Given the well-established role of microbial control in promoting efficient wound healing, these findings indicate that HP and AM EOs could serve as promising natural components in the development of multifunctional wound dressings or bioactive formulations with both antimicrobial and therapeutic benefits.

A comparison with recent studies highlights both consistent trends and distinct contributions regarding the antimicrobial and wound healing potential of these EOs. In the study by Demirhan et al. [1], HP incorporated into chitosan-based hydrogels showed relatively modest antimicrobial activity, with the therapeutic effect mainly attributed to the biomaterial matrix rather than the oil itself. Similarly, Kurt et al. [27] demonstrated that nano-emulsion-based hydrogels enhanced antimicrobial efficacy and significantly improved wound healing outcomes, including re-epithelialization and collagen deposition. In contrast, the present results indicate that HP EO, particularly in combination with AM, exhibits intrinsic antimicrobial activity, especially against Gram-positive bacteria, with MIC values as low as 0.56 μL/mL. This observation is further supported by the findings of Ghasemi et al. [2], who reported that AM EO exhibited bacteriostatic activity against *S. aureus* and *P. aeruginosa* and significantly enhanced wound healing in vivo by increasing collagen content and reducing inflammation. Compared to these studies, our results emphasize that optimizing essential oil combinations can enhance antimicrobial efficacy even in the absence of delivery systems. Collectively, these findings suggest that while advanced formulations improve stability and tissue regeneration, the intrinsic antimicrobial potential of carefully selected essential oil mixtures represents a critical complementary factor for effective wound healing applications.

The interaction between HP and AM EOs, evaluated by means of the fractional inhibitory concentration index (FICI), revealed a strain- and ratio-dependent effect (Table 5). Synergistic activity (FICI ≤ 0.5) was observed exclusively against *S. epidermidis*, particularly for the 60%:40% and 70%:30% combinations (FICI = 0.34). For *S. aureus*, most combinations exhibited additive effects (FICI = 0.70), while the 70%:30% ratio resulted in an indifferent interaction (FICI = 1.48). In contrast, all combinations showed indifferent effects against *S. pyogenes* and *P. aeruginosa* (FICI = 2.00), regardless of the mixing ratio. Similarly, Gram-negative bacteria, including *E. coli* and *K. pneumoniae*, exhibited predominantly indifferent responses (FICI = 1.48–3.10). The antifungal activity against *C. albicans* also showed indifferent interactions across all tested ratios (FICI = 3.10).

**Figure 6:**
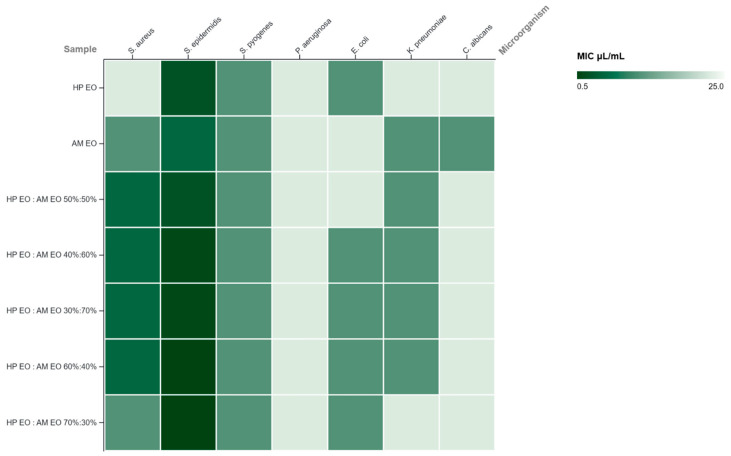
Heatmap representation of the antimicrobial activity of *Hypericum perforatum* (HP) and *Achillea millefolium* (AM) EOs and their combinations against selected microorganisms based on MIC values (µL/mL). Lower MIC values indicate stronger antimicrobial activity.

**Figure 7:**
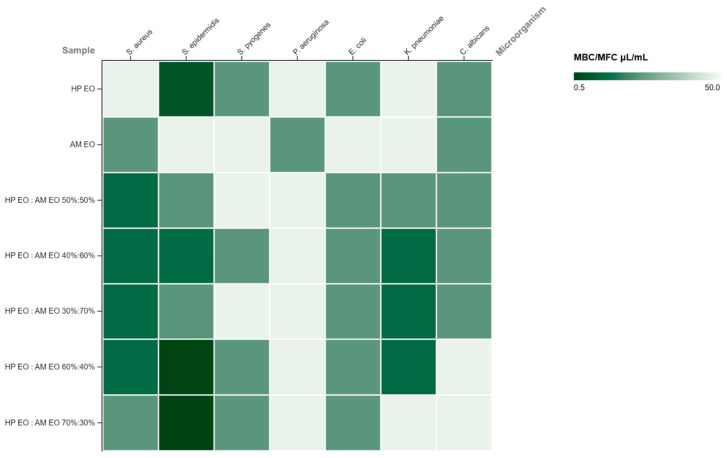
Heatmap representation of the antimicrobial activity of *Hypericum perforatum* (HP) and *Achillea millefolium* (AM) EOs and their combinations against selected microorganisms based on MBC/MFC values (µL/mL). Lower MBC/MFC values indicate stronger antimicrobial activity.

The heatmap visualization of MIC and MBC values provides an intuitive overview of the antimicrobial activity patterns of HP and AM essential oils and their combinations across the tested microorganisms. The MIC heatmap (Figure 6) highlighted distinct susceptibility patterns among the tested microorganisms, confirming greater sensitivity of Gram-positive bacteria, particularly *S. epidermidis*, and the enhanced activity of HP-enriched combinations. The visualization also emphasized the strain-dependent nature of the antimicrobial response, with limited effects against Gram-negative bacteria and *C. albicans*. Similarly, the MBC heatmap (Figure 7) supported that bactericidal effects were strongest for HP-rich mixtures, especially against *S. epidermidis*, whereas Gram-negative strains showed lower susceptibility. These patterns further support that bactericidal efficacy depended on both microorganism type and essential oil ratio.

A more detailed evaluation of the fixed-ratio combinations revealed a clear ratio-dependent trend in antimicrobial activity. Mixtures enriched in HP EO (60%:40% and 70%:30%) consistently exhibited the strongest effects. This indicates that increasing the proportion of HP enhances antimicrobial efficacy, possibly related to its higher content of active constituents, but also could depend on the relative abundance and interactions of shared bioactive constituents present in both oils, particularly β-caryophyllene, together with the increased contribution of β-thujone in HP-enriched mixtures. In contrast, mixtures with higher proportions of AM EO (30%:70% and 40%:60%) did not further improve activity and, in some cases, resulted in only additive or indifferent interactions, suggesting that AM EO alone does not drive the observed synergy. The maintenance of activity in AM-rich combinations also indicates that AM EO contributes to the overall effect, likely through complementary phyto-complex interactions rather than through the action of single constituents alone. The synergistic effects observed exclusively for *S. epidermidis* further highlight the strain-dependent nature of these interactions. Although both *S. epidermidis* and *S. aureus* are Gram-positive bacteria, notable differences in susceptibility were identified. *S. epidermidis* showed significantly higher sensitivity and clear synergistic responses, whereas *S. aureus* exhibited predominantly additive effects. These differences may be related to variations in cell surface organization and biological roles between the two species. It has been reported that *S. epidermidis* is primarily adapted to a commensal lifestyle and is equipped with factors that promote persistence, whereas *S. aureus* possesses a broader repertoire of aggressive virulence determinants, including toxins [28].

Several aspects of the present study warrant consideration when interpreting the results and in defining future research directions. Antimicrobial activity was evaluated under controlled in vitro conditions using reference strains, which provide a standardized and reproducible framework for comparative assessment, although they may not fully capture the variability of clinical strains associated with wound infections.

In addition, while no biofilm model was included, the focus on planktonic cells allows for an initial characterization of antimicrobial potential, which represents a necessary step prior to more complex systems. Given the well-established role of biofilms in chronic wound infections, future studies will benefit from incorporating biofilm-based models to better reflect in vivo conditions and treatment challenges.

The study design was based on a single antimicrobial assay, enabling consistent cross-sample comparison; however, complementary methodological approaches could provide further insights into the mechanisms of action and efficacy. Equally, while cytotoxicity and biocompatibility assessments were beyond the scope of the present work, these evaluations represent indispensable next steps in establishing the translational potential of the investigated essential oils.

Furthermore, the use of fixed-ratio combinations offers a simplified and practical approach to exploring potential synergistic effects, while more detailed investigations at the level of individual compounds and variable ratios would allow a deeper understanding of interaction dynamics.

Collectively, these considerations define several productive directions for future research, encompassing the use of clinical isolates, biofilm-based experimental models, multi-methodological analytical frameworks, and rigorous safety assessments, with the aim of more thoroughly evaluating the therapeutic applicability of these essential oils.

## 4. Materials and Methods

### 4.1. Reagents, Media, and Instrumentation

The media used for antimicrobial activity assays, Tween 80 (density: 1.062 g/cm^3^ at 20 °C), ethanol (96%), and 96-well microplates, were purchased from VWR Chemicals (Radnor, PA, USA). Resazurin sodium salt was obtained from Thermo Fisher Scientific Inc. (Waltham, MA, USA). All microbial strains were acquired from Microbiologics (Saint Cloud, MI, USA), except for Candida albicans, which was provided by the Leibniz Institute (Berlin, Germany). Gentamicin and ketoconazole were purchased from Sigma-Aldrich (Darmstadt, Germany).

The chemical composition of the essential oils was analyzed using a Shimadzu QP 2010 PLUS gas chromatograph–mass spectrometer (GC–MS) equipped with an AOC-20i+s autosampler and a ZB-5MS Plus capillary column (30 m × 0.25 mm i.d., 0.25 μm film thickness; Phenomenex (Torrance, CA, USA)).

### 4.2. Collection of Plant Samples

The inflorescences of HP were collected at full flowering stage in July 2025 from the central Transylvanian region, Romania. The plant material was manually harvested and subsequently dried under natural conditions, protected from direct light exposure, for 14 days.

AM plants were also harvested at full flowering stage. The inflorescences, including approximately 20 cm of the upper stem, were collected from the Târgu Secuiesc area (Covasna County, Romania). The harvested material was subjected to the same drying conditions as described above.

### 4.3. EOs Samples

The essential oils were obtained from dried aerial parts by hydrodistillation using a Neo-Clevenger-type apparatus. Briefly, 100 g of powdered plant material were immersed in 1 L of distilled water and subjected to boiling for 3 h [4,29]. The obtained EO was collected and measured. Yield was calculated as ml of essential oil per 100 g of plant material free of moisture. Then, the samples were stored at refrigerator (4 °C) for further use.

### 4.4. GC–MS Chemical Profiling

The volatile compounds were analyzed by gas chromatography–mass spectrometry (GC–MS). The injector and MS transfer line temperatures were set at 250 °C. The oven temperature program was as follows: initial temperature of 60 °C (held for 1 min), increased to 120 °C at 30 °C min^−1^ (held for 5 min), then to 250 °C at 5 °C min^−1^, and finally to 300 °C at 20 °C min^−1^. Helium (99.9999%, Linde, Hungary) was used as the carrier gas at a constant flow rate of 0.8 mL min^−1^. A volume of 1 µL was injected in split mode (20:1) at 250 °C. Mass spectra were recorded in electron impact mode (EI, 70 eV) over an m/z range of 35–800 with a scan time of 500 ms. Compound identification was performed by comparing the obtained spectra with those from the NIST (versions 127 and 147) and WILEY libraries (match ≥ 90%) and by calculating retention indices (RIs) relative to a homologous series of n-alkanes (C_6_–C_20_). The relative abundance of each compound was expressed as the percentage of its peak area relative to the total ion chromatogram area, as previously described [4,29].

### 4.5. Antimicrobial Activity

#### 4.5.1. Preparation of Microbial Strains

The antibacterial activity was evaluated against *Staphylococcus aureus* ATCC 25923, *Staphylococcus epidermidis* NCTC 11047, *Streptococcus pyogenes* ATCC 19615, *Escherichia coli* ATCC 25922, *Klebsiella pneumoniae* NCTC 13438 and *Pseudomonas aeruginosa* ATCC 27853. Each strain was cultured for 24 h in 10 mL of sterile nutrient broth at 37 °C. Subsequently, a loopful of culture was streaked onto Mueller–Hinton agar plates and incubated at 37 °C for 18–20 h. The bacterial suspensions were adjusted to the turbidity of a 0.5 McFarland standard (approximately 1.5 × 10^8^ CFU/mL) and further diluted to obtain a final inoculum of 10^6^ CFU/mL for microplate assays [4,30].

The antifungal activity was evaluated against *Candida albicans* DSMZ 1386. The strain was cultured in 10 mL of sterile potato dextrose broth (PDB), with incubation at 35 °C for 24 h. Subsequently, a loopful of inoculum was streaked onto potato dextrose agar (PDA) plates and incubated for 18–20 h at 35 °C. Cell suspensions were adjusted to approximately 10^6^ CFU/mL prior to testing [4,31,32].

The morphology of bacterial and fungal cells was confirmed using optical microscopy.

#### 4.5.2. Determination of the Minimum Inhibitory Concentration (MIC)

The minimum inhibitory concentration (MIC) was determined using a resazurin-based microtiter plate assay. EO stock solutions were prepared using a mixture of 50% ethanol and Tween 80 in a 1:8:1 ratio (100 µL EO, 800 µL ethanol 50%, and 100 µL Tween 80), where Tween 80 acted as a non-ionic surfactant to improve EO dispersion in the aqueous medium. The solvent mixture was also included as a negative control to exclude any antimicrobial effect. In a 96-well microtiter plate, 100 µL of sterile nutrient broth (for bacterial assays) or potato dextrose broth (for fungal assays) and 100 µL of EO solution were added to the first well. Serial two-fold (1:1) dilutions were performed across the row by transferring 100 µL from one well to the next, with the final 100 µL discarded. Each well was inoculated with 10 µL of microbial suspension (1.5 × 10^6^ CFU/mL for bacteria and 10^6^ CFU/mL for fungi). Gentamicin (0.04 mg/mL in saline) was used as a positive control for bacterial assays, while ketoconazole (1 mg/mL in saline) served as the positive control for fungal assays. The solvent mixture (ethanol 50% and Tween 80, 1:8:1) was used as a negative control. Microplates were incubated at 37 °C for 22 h (bacteria) and 35 °C for 22 h (yeasts). Following incubation, 20 µL of resazurin solution (0.2 mg/mL) was added to each well and the plates were further incubated for 2 h under the same conditions. The MIC was defined as the lowest EO concentration at which the blue color of resazurin remained unchanged, indicating inhibition of microbial growth. All experiments were performed in duplicate [4,29,33].

The interaction between essential oils was evaluated by calculating the fractional inhibitory concentration (FIC) index based on the MIC values. The FIC for each component was determined as the ratio between the MIC of the essential oil in combination and its MIC when tested individually. The fractional inhibitory concentration index (FICI) was calculated as the sum of the individual FIC values.

Since the essential oils were tested as fixed-ratio combinations, the MIC value of the mixture was used for both components in the calculation. The interactions were interpreted as synergistic (FICI ≤ 0.5), additive (0.5 < FICI ≤ 1), indifferent (1 < FICI ≤ 4), or antagonistic (FICI > 4), according to established criteria [17,34].

$$FICI=\frac{{MIC}_{HP}in\;combination}{{MIC}_{HP}alone}+\frac{{MIC}_{AM}in\;combination}{{MIC}_{AM}alone}$$

#### 4.5.3. Determination of the Minimum Bactericidal Concentration (MBC) and Minimum Fungicidal Concentration (MFC)

The minimum bactericidal concentration (MBC) was determined by subculturing aliquots from the wells showing no visible growth in the MIC assay onto Mueller–Hinton agar plates for bacteria and potato dextrose agar (PDA) plates for Candida albicans. The plates were incubated at 37 °C for bacteria and 35 °C for Candida strain for 24 h. The MBC was defined as the lowest concentration at which no microbial growth was observed. All experiments were performed in duplicate [4,35].

Heatmap analysis was performed to visually assess patterns of antimicrobial activity and to facilitate the comparison of MIC and MBC values across different essential oil treatments and microbial strains. The heatmaps were generated using RAWGraphs (RAWGraphs 2.0), an open-source data visualization platform.

## 5. Conclusions

The present study demonstrates that the antimicrobial activity of HP and AM essential oils is strongly governed by their chemical composition and blending ratio. The volatile profiles identified, dominated by constituents such as caryophyllene, β-thujone, aromadendrene, and chamazulene, are consistent with the biological activity observed. Both oils exhibited higher efficacy against Gram-positive bacteria, particularly *Staphylococcus epidermidis*, as confirmed by MIC, MBC, FICI, and heatmap analyses. Notably, synergistic effects were observed only for combinations enriched in *H. perforatum* (60:40 and 70:30), highlighting the importance of formulation in enhancing antimicrobial performance. The integration of chemical and biological data suggests that the activity of these essential oils is closely related to the presence and interaction of bioactive compounds. These findings support the potential of optimized essential oil combinations as natural antimicrobial agents, particularly for wound management targeting Gram-positive pathogens. Nevertheless, certain limitations of the present study must be acknowledged. The evaluation was confined to in vitro conditions, and the absence of cytotoxicity and biocompatibility data restricts direct extrapolation to clinical settings. Additionally, the use of fixed-ratio combinations does not fully capture the complexity of interactions between individual constituents. Future research should focus on in vivo validation using wound models and on the development of advanced delivery systems, such as hydrogels or nanostructured carriers, to improve stability and enable controlled release. Further elucidation of compound-specific mechanisms and anti-inflammatory effects will be essential to comprehensively establish the therapeutic potential of these essential oil combinations.

## Figures and Tables

**Table 1:** Volatile compounds identified in *Hypericum perforatum* by GC-MS analysis.

No.	Compound	Chemical Class	Retention Time (min)	Relative Content (%)
1	Octane, 2,3,3-trimethyl-	C5	5.524	3.80
2	Nonane	C5	6.184	0.81
3	α-Thujene	C1	6.739	0.28
4	α-Pinene	C1	6.907	1.95
6	3-Octen-5-yne, 2,7-dimethyl-	C5	7.923	9.03
7	β-Myrcene	C1	8.040	2.22
8	α-Fenchene	C1	8.949	4.87
9	cis-β-Ocimene	C1	9.171	1.35
10	γ-Terpinene	C1	9.424	0.25
11	2-Methyldecane	C5	9.506	1.00
13	Undecane	C5	10.206	1.48
14	Linalool	C3	10.398	0.27
15	Cyclohexene derivative	C3	11.384	3.02
16	Pinocamphone	C3	11.570	6.36
17	β-Thujone	C3	12.063	18.47
19	Linalyl anthranilate	C4	13.036	1.76
20	2-Methyldodecane	C5	13.291	1.92
21	Neryl acetate	C4	13.591	0.31
22	2,6,11-Trimethyldodecane	C5	13.901	0.80
23	γ-Elemene	C2	14.545	0.29
24	α-Cubebene	C2	14.817	0.92
25	Longipinene	C2	14.923	0.47
26	Ylangene	C2	15.212	0.47
27	Copaene	C2	15.335	0.69
28	α-Bourbonene	C2	15.507	1.90
29	Caryophyllene	C2	16.311	20.74
30	Germacrene D	C2	16.364	0.69
32	β-Farnesene	C2	16.588	3.49
33	α-Himachalene	C2	16.667	0.39
34	α-Caryophyllene	C2	16.747	0.73
35	Aromadendrene	C2	16.834	0.87
37	Unidentified compound	C6	17.089	0.97
38	α-Amorphene	C2	17.229	1.54
39	γ-Elemene	C2	17.416	1.79
40	Unidentified compound	C6	17.637	0.30
41	δ-Amorphene	C2	17.695	0.47
42	Hedycaryol	C3	18.165	1.48
44	Caryophyllene oxide	C3	18.750	0.68
45	β-Eudesmol	C3	19.777	0.64
46	1-Tetradecanol	C5	19.877	0.20
47	Phytone	C5	22.070	0.12
48	Phytol	C5	25.386	0.22

C1—monoterpene hydrocarbons, C2—sesquiterpene hydrocarbons, C3—oxygenated monoterpenes/oxygenated terpenes, C4—esters, C5—other compounds (such as aliphatic hydrocarbons, alcohols and ketones), C6—unidentified compounds. Relative content (%) was calculated from GC-MS peak areas.

**Table 2:** Volatile compounds identified in *Achillea millefolium* by GC-MS analysis.

No.	Compound	Chemical Class	Retention Time (min)	Relative Content (%)
1	Santolina triene	C1	6.251	0.63
2	α-Thujene	C1	6.742	0.55
3	α-Fenchene	C1	6.914	4.28
4	Camphene	C1	7.245	0.38
5	3-Methylene-bicyclo[3.2.1]oct-6-en-8-ol	C3	7.979	11.41
6	3-Octen-5-yne,2,7-dimethyl-,(E)	C6	8.072	3.19
7	Yomogi alcohol	C3	8.213	0.76
8	α-Terpinolene	C1	8.634	1.35
9	1,8-Cineole	C3	9.022	5.83
10	cis-β-Ocimene	C1	9.181	1.26
11	2-Carene	C1	9.477	2.38
12	Dicyclopropyl carbinol	C6	9.547	0.27
13	3,3,6-Trimethyl-1,5-heptadien-4-ol	C3	9.870	0.35
14	α-Terpinolene	C1	10.002	0.60
15	Linalool	C3	10.248	0.31
16	p-Menth-2-en-1-ol	C3	10.777	0.14
17	Camphor	C3	11.246	0.66
18	Chrysanthemol	C3	11.409	0.99
19	Pinocarvone	C3	11.517	0.17
20	4-Thujanol/Sabinene hydrate	C3	11.973	6.27
21	α-Terpineol	C3	12.169	1.10
22	1-Imidazol-1-yl-3-methylbut-2-en-1-one	C6	12.792	0.15
23	4-Thujen-2-yl acetate	C4	13.413	0.90
24	(-)-trans-Myrtanyl acetate	C4	13.481	0.65
25	Geranyl acetate	C4	13.612	1.11
26	Bornyl acetate	C4	13.715	0.80
27	Pinocarvyl acetate	C4	13.892	0.19
28	γ-Elemene	C2	14.539	0.07
29	δ-Elemene	C2	14.581	0.06
30	α-Terpinyl acetate	C4	14.758	0.28
31	Neryl acetate	C4	14.903	0.16
32	α-Cubebene	C2	15.313	0.21
33	β-Bourbonene	C2	15.482	1.28
34	Caryophyllene	C2	16.248	12.97
35	Germacrene D	C2	16.300	0.23
36	β-Sesquiphellandrene	C2	16.355	0.33
37	cis-α-Bisabolene	C2	16.723	2.21
38	Aromadendrene	C2	17.307	19.12
39	γ-Elemene	C2	17.414	0.96
40	D-Amorphene	C2	17.651	0.29
41	β-Nerolidol	C5	18.220	0.63
42	Caryophyllene oxide	C5	18.728	2.20
43	Epiglobulol	C5	19.282	0.15
44	δ-Cadinol	C5	19.526	0.36
45	α-Cadinol	C5	19.718	0.43
46	Spathulenol	C5	19.910	0.37
47	Tetradecanal	C6	20.560	0.17
48	Chamazulene	C2	21.063	10.13
49	Phytone	C6	22.072	0.14
50	Phytol	C6	25.437	0.39
51	Eicosane	C6	27.470	0.12
52	Heneicosane	C6	29.530	0.09

C1—monoterpene hydrocarbons; C2—sesquiterpene hydrocarbons; C3—oxygenated monoterpenes; C4—esters; C5—oxygenated sesquiterpenes; C6—other/aliphatic compounds. Relative content (%) was calculated from GC-MS peak areas.

**Table 3:** Minimum inhibitory concentration (MIC) of *Hypericum perforatum* (HP) and *Achillea millefolium* (AM) EOs, individually and in combination.

	Gram-Positive Bacteria			Gram-Negative Bacteria			Yeast
	*Staphylococcus aureus*	*Staphylococcus epidermidis*	*Streptococcus pyogenes*	*Pseudomonas aeruginosa*	*Escherichia coli*	*Klebsiella pneumoniae*	*Candida albicans*
	μL/mL	μL/mL	μL/mL	μL/mL	μL/mL	μL/mL	μL/mL
(HP) EO	22.68 ± 0.00	2.45 ± 0.00	10.80 ± 0.00	22.68 ± 0.00	10.80 ± 0.00	22.68 ± 0.00	22.68 ± 0.00
(AM) EO	10.80 ± 0.00	5.14 ± 0.00	10.80 ± 0.00	22.68 ± 0.00	22.68 ± 0.00	10.80 ± 0.00	10.80 ± 0.00
HP EO:AM EO 50%:50%	5.14 ± 0.00	2.45 ± 0.00	10.80 ± 0.00	22.68 ± 0.00	22.68 ± 0.00	10.80 ± 0.00	22.68 ± 0.00
HP EO:AM EO 40%:60%	5.14 ± 0.00	1.17 ± 0.00	10.80 ± 0.00	22.68 ± 0.00	10.80 ± 0.00	10.80 ± 0.00	22.68 ± 0.00
HP EO:AM EO 30%:70%	5.14 ± 0.00	1.17 ± 0.00	10.80 ± 0.00	22.68 ± 0.00	10.80 ± 0.00	10.80 ± 0.00	22.68 ± 0.00
HP EO:AM EO 60%:40%	5.14 ± 0.00	0.56 ± 0.00	10.80 ± 0.00	22.68 ± 0.00	10.80 ± 0.00	10.80 ± 0.00	22.68 ± 0.00
HP EO:AM EO 70%:30%	10.80 ± 0.00	0.56 ± 0.00	10.80 ± 0.00	22.68 ± 0.00	10.80 ± 0.00	22.68 ± 0.00	22.68 ± 0.00
Gentamicina (0.4 mg/mL)/Ketoconazol (1 mg/mL)	0.27 ± 0.00	2.45 ± 0.00	0.13 ± 0.00	0.27 ± 0.00	0.56 ± 0.00	0.27 ± 0.00	0.27 ± 0.00

The values are expressed as mean of two replicates ± SD.

**Table 4:** Minimum bactericidal/fungicidal concentration (MBC/MFC) of *Hypericum perforatum* (HP) and *Achillea millefolium* (AM) EOs, individually and in combination.

	Gram-Positive Bacteria			Gram-Negative Bacteria			Yeast
	*Staphylococcus aureus*	*Staphylococcus epidermidis*	*Streptococcus pyogenes*	*Pseudomonas aeruginosa*	*Escherichia coli*	*Klebsiella pneumoniae*	*Candida albicans*
	μL/mL	μL/mL	μL/mL	μL/mL	μL/mL	μL/mL	μL/mL
(HP) EO	47.62 ± 0.00	5.14 ± 0.00	22.68 ± 0.00	47.62 ± 0.00	22.68 ± 0.00	47.62 ± 0.00	22.68 ± 0.00
(AM) EO	22.68 ± 0.00	47.62 ± 0.00	47.62 ± 0.00	22.68 ± 0.00	47.62 ± 0.00	47.62 ± 0.00	22.68 ± 0.00
HP EO:AM EO 50%:50%	10.80 ± 0.00	22.68 ± 0.00	47.62 ± 0.00	47.62 ± 0.00	22.68 ± 0.00	22.68 ± 0.00	22.68 ± 0.00
HP EO:AM EO 40%:60%	10.80 ± 0.00	10.80 ± 0.00	22.68 ± 0.00	47.62 ± 0.00	22.68 ± 0.00	10.80 ± 0.00	22.68 ± 0.00
HP EO:AM EO 30%:70%	10.80 ± 0.00	22.68 ± 0.00	47.62 ± 0.00	47.62 ± 0.00	22.68 ± 0.00	10.80 ± 0.00	22.68 ± 0.00
HP EO:AM EO 60%:40%	10.80 ± 0.00	1.17 ± 0.00	22.68 ± 0.00	47.62 ± 0.00	22.68 ± 0.00	10.80 ± 0.00	47.62 ± 0.00
HP EO:AM EO 70%:30%	22.68 ± 0.00	0.56 ± 0.00	22.68 ± 0.00	47.62 ± 0.00	22.68 ± 0.00	47.62 ± 0.00	47.62 ± 0.00
Gentamicina (0.4 mg/mL)/Ketoconazol (1 mg/mL)	0.27 ± 0.00	2.45 ± 0.00	0.13 ± 0.00	0.27 ± 0.00	0.56 ± 0.00	0.27 ± 0.00	0.27 ± 0.00

The values are expressed as mean of two replicates ± SD.

**Table 5:** Fractional Inhibitory Concentration Index (FICI) of *Hypericum perforatum* (HP) and *Achillea millefolium* (AM) EOs Combinations.

Combination (HP EO:AM EO)	*S. aureus*	*S. epidermidis*	*S. pyogenes*	*P. aeruginosa*	*E. coli*	*K. pneumoniae*	*C. albicans*
50%:50%	0.70 (Add)	1.48 (Ind)	2.00 (Ind)	2.00 (Ind)	3.10 (Ind)	1.48 (Ind)	3.10 (Ind)
40%:60%	0.70 (Add)	0.71 (Add)	2.00 (Ind)	2.00 (Ind)	1.48 (Ind)	1.48 (Ind)	3.10 (Ind)
30%:70%	0.70 (Add)	0.71 (Add)	2.00 (Ind)	2.00 (Ind)	1.48 (Ind)	1.48 (Ind)	3.10 (Ind)
60%:40%	0.70 (Add)	0.34 (Syn)	2.00 (Ind)	2.00 (Ind)	1.48 (Ind)	1.48 (Ind)	3.10 (Ind)
70%:30%	1.48 (Ind)	0.34 (Syn)	2.00 (Ind)	2.00 (Ind)	1.48 (Ind)	3.10 (Ind)	3.10 (Ind)

Add—additive effect (0.5 < FICI ≤ 1); Ind—indifferent effect (1 < FICI ≤ 4); Syn—synergistic effect (FICI ≤ 0.5).

## Data Availability

The original contributions presented in this study are included in the article. Further inquiries can be directed to the corresponding author.

## Supplementary Materials

The following supporting information can be downloaded at: https://www.mdpi.com/article/10.3390/molecules31101594/s1, Figure S1: Minimum inhibitory concertation of the combination of HP and AM EOs; Figure S2: Minimum bactericidal concertation of combination of HP and AM EOs against Pseudomonas aeruginosa (a) and Klebsiella pneumoniae (b).

## Abbreviations

The following abbreviations are used in this manuscript:

Eos	Essential oils
HP	*Hypericum perforatum*
AM	*Achillea millefolium*
