# Supplementary material for: Chemical Composition and Synergistic Antimicrobial Activity of Hypericum perforatum and Achillea millefolium Essential Oils Against Wound-Associated Microorganisms
Source: Molecules. 2026 May 10;31(10):1594. doi: 10.3390/molecules31101594 (PMC13209759; doi:10.3390/molecules31101594)
Supplement: Supplementary file 1 [file molecules-31-01594-s001.zip › molecules-4264324-supplementary.pdf]

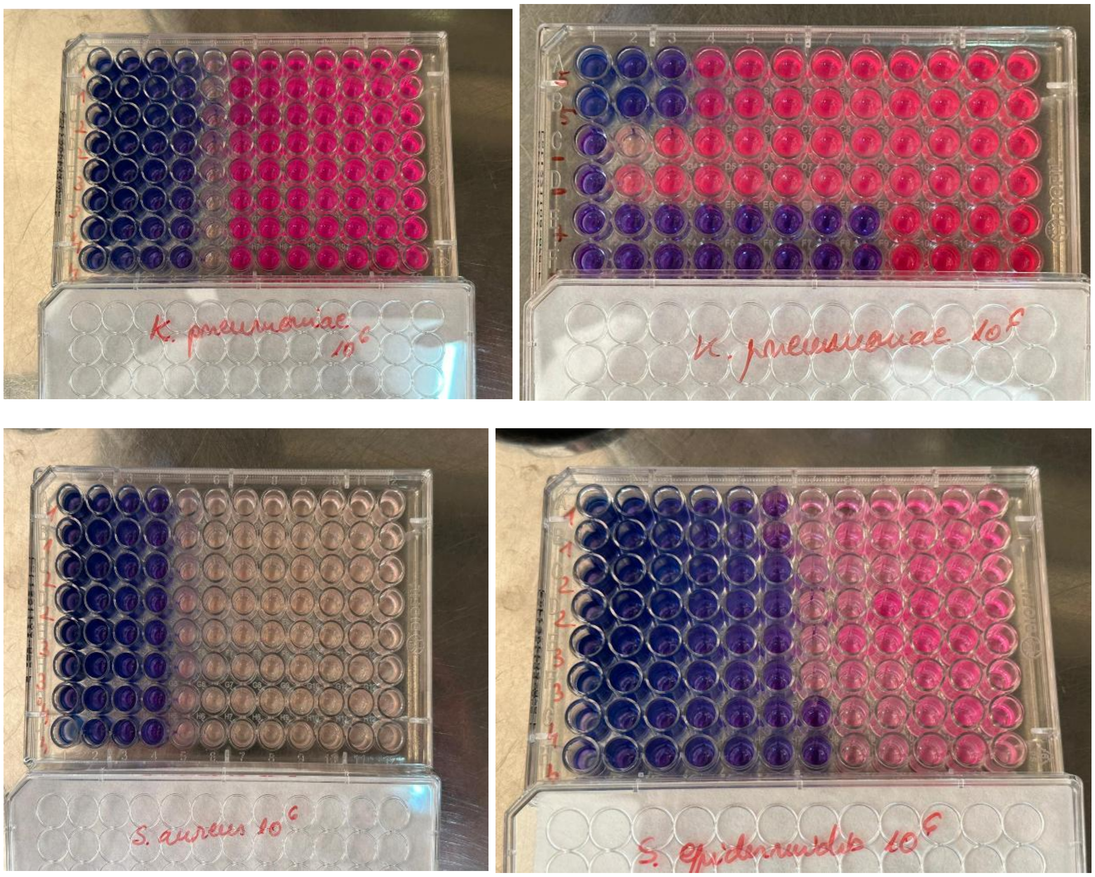

Figure S1. Minimum inhibitory concertation of the combination of HP and AM EOs (1- 50%:50%; 2 – 40%-60%; 3 – 30%-70%; 4 – 60%:40%; 5 – 70%:30%, “+” represents the positive control – gentamicin and “– “represents the negative control – mixture where EO in replace with distillated water)

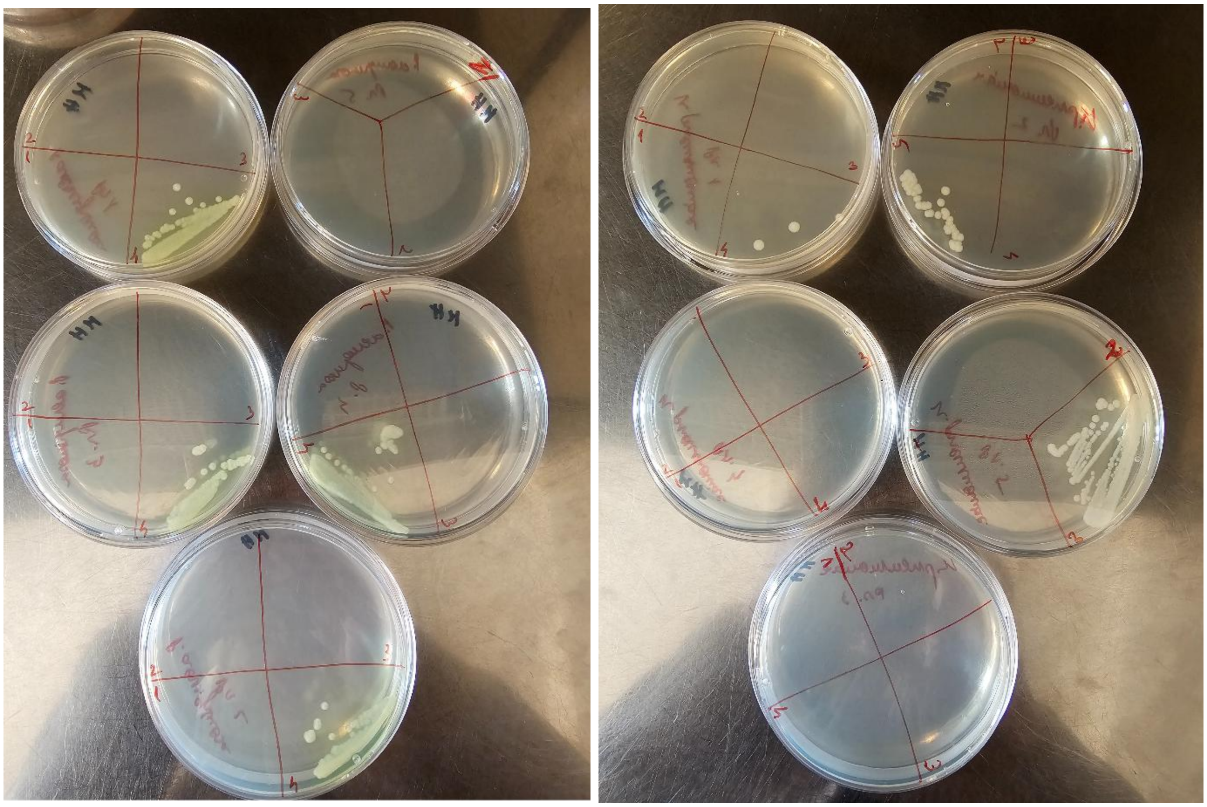

(a) (b)

Figure S2. Minimum bactericidal concertation of combination of HP and AM EOs against *Pseudomonas aeruginosa* (a) and *Klebsiella pneumoniae* (b)
